# Supplementary material for: Analysis of Rac/Rop Small GTPase Family Expression in Santalum album L. and Their Potential Roles in Drought Stress and Hormone Treatments
Source: Life (Basel). 2022 Nov 26;12(12):1980. doi: 10.3390/life12121980 (PMC9787843; doi:10.3390/life12121980)
Supplement: Supplementary file 1 [file life-12-01980-s001.zip › Table S1.pdf]

Table S1. Primer design

Gene name	Forward primer (5′- 3′)	Reverse primer (5′- 3′)
Actin	TGCTCTTCCCCATGCCAT	AACAATTTCCCGCTCAGCAG
SaRac1	ACCAGCAACACATTTCCCAC	TGTTGTAGTCCTCCTGTCCG
SaRac2	TACAGGGGTGCAGATGTGTT	CTACGAGCACAATTGGCACA
SaRac3	TAAGTTATCGCGGGGCAGAT	GGTCAAGCTTCGTTCCAACA
SaRac4	GACCCACCCGATTGAGAGAA	AGGCTGAGAGAGAGAGGGAA
SaRac5	GCTCGATTCATCAAGTGCGT	AACACAGCCATCTACCACCA
SaRac6	GCCCATTTCTTCAGCTCAGG	AGCACAACCTTAATCGCTGC
SaRac7	TGTCAATTTGGGGCTTTGGG	CAGGCATCCACTTCTTGAGC
SaRac8	AAGGCCCTTAAGCTACCGAG	GTGCCAGCCAATACAAGAGG
SaRac9	CCAATCACAACAGCTCAGGG	CCTTAATGGCCGCATCGAAA
