# Supplementary material for: Analysis of Rac/Rop Small GTPase Family Expression in Santalum album L. and Their Potential Roles in Drought Stress and Hormone Treatments
Source: Life (Basel). 2022 Nov 26;12(12):1980. doi: 10.3390/life12121980 (PMC9787843; doi:10.3390/life12121980)
Supplement: Supplementary file 1 [file life-12-01980-s001.zip › Table S2.pdf]

Table S2. Segmental replication events in sandalwood

Gene name1	Gene name2
Sal7G05910.1	Sal10G07090.1
Sal7G05910.1	Sal8G02490.1
Sal8G02490.1	Sal10G07090.1
Sal9G04490.1	Sal10G07090.1
Sal9G09920.1	Sal10G07090.1
