# Supplementary material for: Analysis of Rac/Rop Small GTPase Family Expression in Santalum album L. and Their Potential Roles in Drought Stress and Hormone Treatments
Source: Life (Basel). 2022 Nov 26;12(12):1980. doi: 10.3390/life12121980 (PMC9787843; doi:10.3390/life12121980)
Supplement: Supplementary file 1 [file life-12-01980-s001.zip › Table S3.pdf]

**Table S3.** Synteny analysis of Rac genes between sandalwood, *A. thaliana* and rice.

| Species | Gene ID | Gene ID (sandalwood) | The positon of the genes |
|---|---|---|---|
| *Arabidopsis thaliana* | AT1G75840.1<br>AT2G17800.1<br>AT3G51300.1<br>AT4G35950.1 | Sal10G07090.1 | 5966510 to 5969147bp |
| | AT1G20090.1<br>AT1G75840.1<br>AT2G17800.1 | Sal8G02490.1 | 2009257 to 2011676bp |
| | AT1G75840.1<br>AT1G20090.1<br>AT4G35020.3 | Sal7G05910.1 | 4738277 to 4742259bp |
| | AT5G45970.1 | Sal9G31620.1 | 27951102 to 27954702bp |
| | AT1G75840.1<br>AT2G17800.1<br>AT3G51300.1<br>AT4G35950.1 | Sal9G09920.1 | 7927365 to 7929192bp |
| | AT4G28950.1 | Sal6G04230.1 | 3056752 to 3059680bp |
| | AT5G62880.1 | Sal9G07020.1 | 5777602 to 5782565bp |
| | AT1G20090.1<br>AT1G75840.1<br>AT2G17800.1<br>AT4G35020.3 | Sal9G04490.1 | 3684651 to 3689159bp |
| *Oryza sativa L.* | Os02t0120800-02 | Sal7G05910.1 | 4738277 to 4742259bp |
| | Os01t0229400-02 | Sal6G04230.1 | 3056752 to 3059680bp |
| | Os02t0742200-02 | Sal9G07020.1 | 5777602 to 5782565bp |
